# Premature ejaculation among Chinese urban men: prevalence and correlates

**DOI:** 10.1093/sexmed/qfac015

**Published:** 2023-01-12

**Authors:** Caoyuan Niu, Daniel Ventus, Patrick Jern, Pekka Santtila

**Affiliations:** School of Psychology and Cognitive Science, East China Normal University, Shanghai 200062, People’s Republic of China; Faculty of Arts and Sciences, NYU Shanghai, Shanghai 200122, People’s Republic of China; Experience Lab, Faculty of Education and Welfare Studies, Åbo Akademi University, Turku FI-20500, Finland; Faculty of Arts, Psychology and Theology, Åbo Akademi University, Turku FI-20500, Finland; School of Psychology and Cognitive Science, East China Normal University, Shanghai 200062, People’s Republic of China; Faculty of Arts and Sciences, NYU Shanghai, Shanghai 200122, People’s Republic of China

**Keywords:** premature ejaculation, sexual experience, frequency of sex, erectile function

## Abstract

**Background:**

Premature ejaculation (PE) is a common male sexual dysfunction worldwide. It leads to substantial distress in men and their partners, constitutes a serious threat to the quality and stability of romantic relationships, and results in a decreased quality of life in a large part of the population.

**Aim:**

We investigated the prevalence of PE and correlated factors in an urban sample of Chinese men.

**Methods:**

In total, 1976 Chinese men aged 18 to 50 years responded to an online questionnaire regarding background information, present and previous sexual experience, frequency of different types of sex, as well as erectile and ejaculatory function.

**Outcomes:**

Participants’ age, assigned sex at birth, sexual identity, relationship status, present and previous sexual experience, frequency of sexual activities, International Index of Erectile Function–5, and Checklist for Early Ejaculation Symptoms were used in the analyses.

**Results:**

Forty-four (2.3%) participants had scores that were indicative or strongly indicative of PE, which was highly correlated with erectile problems. Men with more sexual experience (ie, more sexual partners and longer duration of being sexually active) had fewer ejaculatory problems. More frequent masturbation was associated with ejaculatory problems when controlling for age and education. More frequent partnered sex (ie, penile-vaginal sex) was associated with fewer ejaculatory problems. Ejaculation latency times for different types of sexual activities were positively correlated.

**Clinical Translation:**

The results indicated that ejaculatory problems have complex relationships with sexual experience that clinicians should be aware of.

**Strengths and Limitations:**

This study was the first to investigate PE with the Checklist for Early Ejaculation Symptoms as the measurement tool and the associations between PE and sexual experience, frequency of sexual activities, and sexual function in a large Chinese sample. However, self-reported ejaculation latency times may suffer from problems with validity.

**Conclusion:**

Men’s sexual experience (ie, more sexual partners and longer duration of being sexually active) has an effect on their sexual function, which in turn affects their sexual activity.

## Introduction

Premature ejaculation (PE) is a common sexual dysfunction for men worldwide. It is characterized by a lack of control over the timing of ejaculation, a short ejaculation latency time (ie, the time from penetration to ejaculation), and subsequent sexual distress. While the proportion of men in international studies who fulfill all diagnostic criteria, including a latency time <1 minute, is about 3%,^1^ approximately 1 in 4 men reports experiencing distress due to his ejaculation commencing more rapidly than he would like to. A recent review indicated that the prevalence of men affected by PE ranged from 20% to 75%.^2^ According to the International Society for Sexual Medicine, PE can be classified into 2 types: lifetime PE and acquired PE. Lifetime PE is defined as ejaculation always or almost always occurring before vaginal penetration or within about 1 minute of the first penetration sexual. Acquired PE is defined as the ejaculation time having a clinically significant and distressing reduction later in life, including an inability to delay ejaculation beyond about 3 minutes.^3^ In contrast, delayed ejaculation is an inhibition of ejaculatory reflex, which ranges from increases in ejaculatory latency to a complete inability to ejaculate and can also lead to distress and interpersonal difficulties.^4^ The prevalence of delayed ejaculation ranges from 1% to 4%,^4^ and its likelihood increases with age and for those with diabetes.^5^

The etiology of PE is poorly understood.^1^ The risk factors for PE include psychological and biological factors—for example, performance anxiety and negative early sexual experiences,^2^ as well as diabetes mellitus and obesity.^6^

Research has shown that PE is associated with a range of negative outcomes, including low self-confidence and self-esteem,^7^^,^^8^ anxiety,^9^ interpersonal difficulties,^10^^,^^11^ and depression.^9^^,^^12^ The partner of a man with PE is often affected negatively as the man himself.^7^^,^^12^ In fact, partners of men with PE report lower relationship and sexual satisfaction, as well as an increased prevalence of sexual dysfunctions.^13^^,^^14^ In a similar vein, a large cross-cultural study found that 22% of women reported that a man’s ejaculatory problems had been grounds for a breakup or divorce.^15^ Taken together, it is clear that PE leads to substantial distress in men and their partners, constitutes a serious threat to the quality and stability of romantic relationships, and consequently results in a decreased quality of life in a large part of the population.^7^^,^^12^^,^^16^

Erectile dysfunction (ED) is the inability to attain or maintain a penile erection sufficient to obtain satisfaction from sexual intercourse.^17^ ED is also a common sexual dysfunction worldwide and has a negative effect on self-esteem and sexual satisfaction.^18^^,^^19^ ED primarily occurs in men aged >40 years.^20^ A meta-analysis found that the prevalence of ED increased from 20.9% in men aged <30 years to 93.7% in men >70 years in mainland China.^21^ The diagnostic methods included self-reports, the Chinese Index of Erectile Function, and the International Index of Erectile Function–5 (IIEF-5). In addition, studies that used the IIEF-5 to measure ED reported the prevalence of mild, moderate, and severe ED as 32.5%, 9.9%, and 14.0%, respectively, with an overall prevalence of 49.9%.^21^

A number of studies have reported that the presence of PE is associated with a 4-fold increased risk of ED independent of the definition used.^22–25^ The higher-risk ED in men with PE is more likely the older the man is.^24^ Additionally, anxiety, depression, and diabetes mellitus have been found to be robustly associated with a higher risk of ED.^24^ A cross-sectional study noted that 77.8% and 34.2% of men with lifelong and acquired PE, respectively, indicated the presence of ED symptoms after the occurrence of PE symptoms.^26^ However, 22.2% and 52.2% men with lifelong and acquired PE stated that the symptoms of PE and ED coexisted.^26^ There is also some evidence suggesting that men with ED may aim to expedite sexual intercourse and experience PE because of the difficulty in maintaining erection and worrying about the short time of sufficient erection.^27^ In addition, some risk factors have been associated with PE and ED: diabetes mellitus, arterial hypertension, obesity, and smoking.^24^ Yet, the underlying mechanism of the relationship between PE and ED has still not been clarified. A large-scale epidemiologic Chinese face-to-face interview study revealed that approximately 34.62% of men complained of PE.^28^ According to the participants’ self-reported intravaginal ejaculatory latency times (IELTs), 11.2% were diagnosed with lifelong or acquired PE.^28^ In recent years, interest in the etiology of PE has increased. So far, few studies have investigated the prevalence of PE via a diagnostic tool in Chinese samples. The aim of the present study was to explore the prevalence of PE and the demographic factors and frequency of different types of sexual activity that are associated with this sexual dysfunction among urban Chinese men. We wanted to answer the following questions: What is the prevalence of PE? How is it associated with age, previous sexual experience, and frequency of different types of sexual activities among urban Chinese men?

### Hypotheses

Higher age is positively linked to a higher risk of ED.^20^^,^^24^ However, previous research suggests that older men have longer self-reported ejaculation latency times.^13^ Consequently, we expected older men to have more erectile but fewer ejaculatory problems. Smoking and cardiovascular disease are risk factors of PE and ED.^24^ We expected that men with a smoking habit or cardiovascular disease would have more erectile and ejaculatory problems.

Previous research has confirmed that performance anxiety during sexual activity may be related to a higher risk of PE and ED.^29^^,^^30^ Individuals who have less sexual experience or negative sexual experiences might have more performance anxiety when they have sexual intercourse with their partners, leading to sexual dysfunctions.^30^^,^^31^ Alternatively, preexisting sexual dysfunctions might lead to avoidance of sexual experiences.^7^ Therefore, we expected that men with less sexual experience would have more erectile and ejaculatory problems.

Men experience less sexual distress^32^ and sexual dysfunction^33^ (PE, ED, or delayed ejaculation) during masturbation as compared with during partnered sex. This result could be related to less performance anxiety during masturbation. This would lead to an expectation of the ejaculatory latency times (ELTs) of penile-vaginal sex to correlate positively with the ELTs of other types of partner sex but not necessarily be associated with the ELTs of masturbation. Previous research has reported positive associations between ELTs of different types of partnered sex.^34^ However, in this research, longer ELTs during masturbation also had a positive association with longer ELTs of penile-vaginal sex. Corty’s finding of a positive association between penile-vaginal intercourse and masturbatory ELTs suggests an etiology unrelated to the social vs nonsocial context.^34^ For example, previous research has found evidence of genetic effects influencing ELTs.^35^ It is therefore not possible to formulate a clear hypothesis about an association between the ELTs for partnered sex and masturbation.

Men with more erectile and ejaculatory problems indicate less sexual satisfaction during partnered sex.^7^^,^^18^ This could lead them to seek sexual satisfaction from masturbation as a compensatory response.^36–38^ Therefore, we expected men who have more erectile and ejaculatory problems to masturbate more frequently, whereas we expected men who have fewer erectile and ejaculatory problems to have partnered sex more frequently.

## Methods

### Participants

The target population of the present study was adult men currently living in the city of Shanghai, China. The age of the participants (N = 1976) ranged from 18 to 50 years (mean = 31.54, SD = 5.27). Initially, 2051 participants completed the survey. The final sample of 1976 participants reported that their biological sex at birth was male, that they were sexually attracted only to women, that their sexual identity was straight, and that they had a stable sexual partner. We wanted to initially focus on straight men with female partners to be able to investigate PE and ED without confounding related to same sex–attracted men who were in relationships with women, a relatively common occurrence in China.^39^

### Measures

#### Demographic information

Participants were first asked to answer questions regarding their age, assigned sex at birth, sexual identity, and if they had a stable sexual partner or not. The response options for sexual identity were *straight*, *gay*, *bisexual*, *asexual*, and *unsure*. They also answered a question about the sex of the persons to whom they were attracted with the response options *men*, *women*, *both*, *neither*, and *unsure*.

Participants were asked to answer questions about the presence of cardiovascular disease and their smoking status: “Do you have or any history of cardiovascular or endocrine disease?” and “Do you smoke?”

#### Present and previous sexual experience

If participants stated that they had a stable sexual partner, they answered questions about the duration of the present relationship and about being sexually active in it. The first question was “How long have you been (sexually active) in your present relationship?” The response options were *less than a month*, *more than a month but less than 6 months*, *7*-*12 months*, *1*-*3 years*, *4*-*10 years*, and *more than 10 years*. They also answered the question “How often on average do you have sexual (penile-vaginal or penile-anal) intercourse with your current partner?” The response options were *less than once a month*, *once a month*, *once every 2 weeks*, *once a week*, *twice a week*, *3 to 4 times a week*, *once a day*, and *more than once a day*.

Participants indicated the longest time that they had been sexually active and the number of sexual partners that they had had. The first question was “What is the longest time you have been sexually active (penile-vaginal or penile-anal sex) with a partner?” The response options for questions were *less than a month*, *more than a month but less than 6 months*, *7-12 months*, *1-3 years*, *4-10 years*, and *more than 10 years*. The second question was “How many sexual partners with whom you have engaged in penile-vaginal or penile-anal intercourse have you had in total?” Here, the participants cited the number of their sexual partners.

#### International Index of Erectile Function–5

The IIEF-5, which was developed from the 15-item version,^40^ was used to measure ED.^41^ A sample item is “How do you rate your confidence that you could get and keep an erection?” The sum of the 5 items was computed so that each item was evaluated on a 5-point Likert scale ranging from 1 to 5 for each participant. In a previous Chinese study, the internal consistency assessed with Cronbach α was 0.790.^42^ The electronic version of the IIEF-5 has excellent internal consistency, test-retest reliability, and convergent validity in Western samples.^43^ Cronbach α was 0.784 for these items in the present study.

There are 5 categories of ED based on IIEF-5 scores: severe (5-7), moderate (8-11), mild to moderate (12-16), mild (17-21), and no ED (22-25).^40^

#### Checklist for Early Ejaculation Symptoms

The Checklist for Early Ejaculation Symptoms (CHEES) is 5-item diagnostic tool developed from 3 earlier ejaculation diagnostic tools and has improved validity; it was used to measure the ejaculatory function of the participants. This measure has been proven to perform well in differentiating men with and without PE in a Western population sample^44^ (area under the curve = 0.98) and to have high reliability (0.760).^45^ It has not previously been used with Chinese samples. Therefore, we first translated the scale into a Chinese version using translation software. This version was then modified by native Chinese-speaking PhD students of human sexuality. A sample item is “Over the past 6 months, what was your control over ejaculation during sexual intercourse?” The response options were as follows: 1 = *very good*, 2 = *good*, 3 = *fair*, 4 = *poor*, 5 = *very poor*. The sum of the 5 items was computed in which each item was evaluated on a 5-point Likert scale ranging from 1 to 5. In the present study, the Cronbach α was 0.776.

A score ranging from 21 to 25 is strongly indicative of fulfilling diagnostic criteria for PE; a score from 17 to 20, indicative of PE; and a score from 5 to 16, a low probability of PE. However, in addition to looking at the summary score, we separately examined the final question regarding latency time, as the updated *DSM-V* and International Society for Sexual Medicine criteria for PE^46^^,^^47^ identify a cutoff of 1 minute as a prerequisite for diagnosis.

Next, the participants answered the question “Think about the entire history of your sexual life. Has your ejaculation always or nearly always occurred prior to or within about 1 minute of vaginal penetration?” If they chose “no,” they would answer the question “Think about the entire history of your sexual life. Has your ejaculation changed so that it used to take you longer but that it now happens prior to or within about 1 minute of vaginal penetration?”

#### Frequency of sexual activities

Participants reported the frequency of masturbation alone while using or not using pornographic materials, masturbation in the presence of their partners, masturbation with manual stimulation by their partners, oral sex, and penile-vaginal and penile-anal sex during the last month. Sample items are “Did you masturbate alone without consuming pornography during the last month?” and “Did you vaginally penetrate your partner during the last month?” For each item, the participants could choose 1 of these response options: *no*, *once*, *twice*, *3-5 times*, *6-10 times*, *11-20 times*, *21-30 times*, and *more than once a day*. If they had engaged in a particular sexual activity during the last month, they were asked a follow-up question regarding how much time usually elapsed between when they started this sexual activity and when they first ejaculated. A sample item is “On average, during vaginal penetration of your partner, how much time elapses between when you first enter your partner vaginally with your penis and when you first ejaculate?” The response options were as follows: *I usually do not ejaculate in this situation*, *more than 10 min*, *between 5 and 10 min*, *between 1 and 5 min*, *less than 1 min*, and *before inserting the penis fully inside the vagina*.

### Procedure

The questionnaire was created on 2 Chinese survey platforms: WJX (https://www.wjx.cn) and CREDAMO (https://www.credamo.com). On both platforms, an invitation link to the questionnaire was sent to potential participants, who were adult men aged 18 to 50 years and currently living in Shanghai (https://www.wjx.cn/vj/reLdx3e.aspx; https://www.credamo.com/survey.html#/share/a3a55918c4d245b5b1486dd24cc23db0). Men who were interested in participating could click the survey link. After they did so, they would read the consent form online to decide whether they wanted to participate in the study and then click “Yes, I agree to participate (and confirm that I am eligible for this study)” in case they did. Only participants who were aged >18 years, male, and Chinese and had a regular sexual partner were asked to participate. The present study was reviewed and approved by the NYU Shanghai Institutional Review Board (2021-054-NYUSH-Zhongbei). Each participant responding on WJX was paid 4 to 7 RMB for their participation while each participant responding on CREDAMO was paid 12.5 RMB, in line with the internal processes of the 2 platforms for a questionnaire of this length. Finally, there were 1055 responses from WJX and 996 from CREDAMO.

Participants from CREDAMO had a higher age (*t*_1974_ = 6.394, *P* < .001) and educational level (*t*_1974_ = 5.706, *P* < .001) than those from WJX. For this reason and because these 2 variables were related to the sexual dysfunction variables, we included them as covariates in the regression analyses. We also investigated the stability of the associations between the sexual dysfunction variables and the other variables between the platforms. After correction for the number of tests, the confidence intervals of correlations from CREDAMO and WJX did not overlap between the CHEES and (1) the length of time in the sexually active present relationship, (2) the number of sexual partners, (3) the frequency of manual masturbation by the partner, and (4) the frequency of penile-anal sex; likewise, they did not overlap between the IIEF-5 and the frequency of manual masturbation by the partner. All other correlations’ confidence intervals overlapped between the platforms.

### Statistical analyses

We used SPSS version 25.0 (IBM) to conduct the data analyses. We conducted bivariate Pearson correlation analyses to explore the relationships among age, education level, erectile function, ejaculatory function, present and prior sexual experience, and frequency of different types of sex. We conducted *t*-tests to investigate the differences in age and educational levels between participants from CREDAMO and WJX. We also conducted bivariate correlations with confidence intervals to explore whether there were differences in the correlations among age, education level, erectile function, ejaculatory function, present and prior sexual experience, and frequency of different types of sex between participants from CREDAMO and WJX. Then, we conducted a series of linear regression analyses to explore if the sexual experience variables had associations with erectile and ejaculatory function after controlling for confounding factors. Next, we conducted a series of linear regression analyses to explore if the frequency of different types of sexual activities had associations with erectile and ejaculatory function after controlling for confounding factors. Then, we conducted a bivariate Person correlation analysis to explore the relationships among the ELTs for different types of sex. However, not all participants engaged in all types of sex. Therefore, the sample size varied in these analyses, while for all other analyses, all participants were always included. Finally, we conducted a *t*-test analysis to explore the difference between the cardiovascular disease groups (have vs not) and smoking status groups (smoke or not) in erectile and ejaculatory function. We also conducted partial correlation to explore the association between erectile and ejaculatory function after controlling for confounding factors.

## Results

Table 1 shows the prevalence of different levels of sexual dysfunction. There were 9 (0.5%) participants whose scores were strongly indicative of PE, 35 (1.8%) whose scores were indicative of PE, and 1932 (97.8%) whose scores suggested a low probability of PE. Only 7 (0.4%) of the 9 participants whose total score was strongly indicative of PE reported that the ELT is <1 minute. Just 2 participants stated that they ejaculated before the penis was inside the vagina. In addition, 8 (0.4%) participants indicated that they do not ejaculate during intercourse. There were also 8 (0.4%) participants with likely moderate ED, 98 (5.0%) with mild to moderate ED, 546 (27.6%) with mild ED, and 1324 (67.0%) with no ED symptoms.

**Table 1:** Prevalence of PE and ED in the sample of urban Chinese men.

**PE** ^**a**^	**No.**	**%**	**ED** ^**b**^	**No.**	**%**
Strongly indicative	9	0.5	Moderate	8	0.4
Indicative	35	1.8	Mild to moderate	95	5.0
Low probability	1932	97.8	Mild	546	27.6
			No ED	1324	67.0

Abbreviations: ED, erectile dysfunction; PE, premature ejaculation.

^a^The Checklist for Early Ejaculation Symptoms was used to measure the ejaculatory function of the participants. A score ranging from 21 to 25 is strongly indicative of fulfilling diagnostic criteria for PE; a score from 17 to 20, indicative of PE; and a score from 5 to 16, a low probability of PE.

^b^The International Index of Erectile Function–5 was used to measure ED. There are 5 categories of ED based on its scores: severe (5-7), moderate (8-11), mild to moderate (12-16), mild (17-21), and no ED (22-25).

Tables 2 and 3 show the frequency of the individual response options for variables related to PE. As presented in Table 3, 57 (2.9%) participants reported that their ejaculation always or nearly always occurred prior to or within about 1 minute of vaginal penetration (ie, lifetime PE), and 39 (2.0%) identified change such that it used to take longer but that it now happened prior to vaginal penetration (ie, anteportal ejaculation) or within 1 minute of it (acquired PE).

**Table 2:** Frequency of response options in the Checklist for Early Ejaculation Symptoms.

	**Response option, No. (%)**					
**Question: Over the past 6 months** ^**a-e**^	**1**	**2**	**3**	**4**	**5**	**6**
1: Was your control over ejaculation during penile-vaginal sexual intercourse?	531 (26.9)	1019 (51.6)	333 (16.9)	84 (4.3)	9 (0.5)	
2: To what extent does how fast you ejaculate during penile-vaginal sexual intercourse cause difficulty in your relationship with your partner?	1009 (51.1)	776 (39.3)	148 (7.5)	37 (1.9)	6 (0.3)	
3: Do you ejaculate with very little stimulation?	1198 (60.6)	588 (29.8)	120 (6.1)	52 (2.6)	18 (0.9)	
4: Do you feel frustrated because of ejaculating before you want to?	680 (34.4)	985 (49.8)	187 (9.5)	99 (5)	25 (1.3)	
5: How much time elapses between when you first enter your partner vaginally with your penis and when you first ejaculate?	8 (0.4)	1388 (70.2)	452 (22.9)	112 (5.7)	14 (0.7)	2 (0.1)

^a^Question 1: 1 = very good, 2 = good, 3 = fair, 4 = poor, 5 = very poor.

^b^Question 2: 1 = not at all, 2 = a little bit, 3 = moderately, 4 = quite a bit, 5 = extremely.

^c^Question 3: 1 = almost never or never, 2 = less than half the time, 3 = about half the time, 4 = more than half the time, 5 = almost always or always.

^d^Question 4: 1 = not at all, 2 = a little bit, 3 = moderately, 4 = quite a bit, 5 = extremely.

^e^Question 5: 1 = usually do not ejaculate, 2 = more than 10 min, 3 = between the 5 and 10 min, 4 = between the 1 and 5 min, 5 = less than 1 min, 6 = before the penis is inside.

**Table 3:** Frequency of response options in questions for the ejaculatory time of entire sexual life.

	**Response option, No. (%)**	
**Think about the entire history of your sexual life . . .** ^**a**^	**Yes**	**No**
1: Has your ejaculation always or nearly always occurred prior to or within about 1 min of vaginal penetration?	57 (2.9)	1919 (96.1)
2: Has your ejaculation changed so that it used to take you longer but that it now happens prior to or within about 1 min of vaginal penetration?	39 (2.0)	1880 (98.0)

^a^Participants would answer the second question only if answering “no” to the first.

Table 4 shows the results of correlation analyses among the demographic factors, current and previous sexual experiences, and ejaculatory and erectile function. First, more erectile problems had a strong association with more ejaculatory problems. The result also showed that higher age and education level were associated with fewer ejaculatory and erectile problems. Longer relationship duration and sexually active time in present and previous relationships and more sexual partners were associated with fewer ejaculatory and erectile problems.

**Table 4:** Correlations among demographic factors, present and previous sexual experience, ejaculatory problems, and erectile function.^a^

	**Ejaculatory problems** **(CHEES)**	**Erectile function** **(IIEF-5)**	**1**	**2**	**3**	**4**	**5**	**6**
Erectile function (IIEF-5)	−0.580^**^							
1: Age	−0.076^**^	0.056^*^						
2: Education	−0.097^**^	0.046^*^	−0.006					
3: Length of present relationship	−0.150^**^	0.213^**^	0.557^**^	0.028				
4: Length of sexual activity in present relationship	−0.193^**^	0.254^**^	0.581^**^	0.030	0.873^**^			
5: Longest time being sexually active	−0.234^**^	0.286^**^	0.569^**^	0.040	0.797^**^	0.877^**^		
6: No. of sexual partners	−0.252^**^	0.212^**^	0.137^**^	0.137^**^	0.096^**^	0.140^**^	0.207^**^	
7: Frequency of penile-vaginal sex	−0.265^**^	0.303^**^	−0.039	0.072^**^	0.026	0.127^**^	0.196^**^	0.247^**^

Abbreviations: CHEES, Checklist for Early Ejaculation Symptoms; IIEF-5, International Index of Erectile Function–5.

^a^Higher CHEES values suggest more ejaculatory problems. Higher IIEF-5 values suggest fewer erectile problems.

^*^P < .05.

^**^ *P* < .01.

Table 5 shows the result of linear regression analyses between current and previous sexual experience and ejaculatory function before controlling for age and education (*R*^2^ = 0.129, *F*_5,1970_ = 59.677, *P* < .001) and after (*R*^2^ = 0.134, *F*_7,1968_ = 44.506, *P* < .001). Table 6 shows the result of linear regression analyses between current and previous sexual experiences and erectile function before controlling for age and education (*R*^2^ = 0.156, *F*_5,1970_ = 73.922, *P* < .001) and after (*R*^2^ = 0.166, *F*_7,1968_ = 57.022, *P* < .001). The results indicated that the longer the participants had been sexually active in a previous relationship and the more sexual partners they had overall had an association with fewer ejaculatory and erectile problems.

**Table 5:** Linear regression analyses between present and previous sexual experience and ejaculatory problems, before and after controlling for age and education.^a^

**CHEES (DV): controlled for**	***B***	**β**	***t***	***P* value**
Length of present relationship	0.026	0.009	0.206	.836
Age, education	0.013	0.005	0.103	.918
Length of sexual activity in present relationship	−0.058	−0.024	−0.439	.660
Age, education	−0.090	−0.037	−0.68	.497
Longest time being sexually active	−0.355	−0.147	−3.225	.001
Age, education	−0.387	−0.160	−3.483	.001
No. of sexual partners	−0.227	−0.172	−7.823	<.001
Age, education	−0.225	−0.171	−7.705	<.001
Frequency of penile-vaginal sex	−0.420	−0.191	−8.501	<.001
Age, education	−0.397	−0.180	−7.942	<.001

Abbreviations: CHEES, Checklist for Early Ejaculation Symptoms; DV, dependent variable.

^a^Upper rows present the results of regression analyses before controlling for age and education; lower rows, after controlling for age and education.

**Table 6:** Linear regression analyses between present and previous sexual experience and erectile function, before and after controlling for age and education.^a^

**IIEF-5 (DV): controlled for**	***B***	**β**	***t***	***P* value**
Length of present relationship	0.120	0.046	1.045	.296
Age, education	0.165	0.063	1.442	.149
Length of sexual activity in present relationship	0.104	0.046	0.863	.388
Age, education	0.173	0.078	1.441	.150
Longest time being sexually active	0.308	0.138	3.077	.002
Age, education	0.387	0.173	3.832	<.001
No. of sexual partner	0.137	0.113	5.199	<.001
Age, education	0.148	0.122	5.592	<.001
Frequency of penile-vaginal sex	0.492	0.241	10.928	<.001
Age, education	0.453	0.222	9.976	<.001

Abbreviations: DV, dependent variable; IIEF-5, International Index of Erectile Function–5.

^a^Upper rows present the results of regression analyses before controlling for age and education; lower rows, after controlling for age and education.

Table 7 shows the results of correlation analyses between the frequency of sex and ejaculatory and erectile function. The results indicated that a higher frequency of any type of masturbation had an association with more ejaculatory and erectile problems. The frequency of oral sex and penile-vaginal sex had an association with fewer ejaculatory and erectile problems. A higher frequency of penile-anal sex had an association only with fewer ejaculatory problems.

**Table 7:** Correlations among the frequency of different types of sex, ejaculatory problems, and erectile function.^a^

	**Ejaculatory problems (CHEES)**	**Erectile function (IIEF-5)**	**1**	**2**	**3**	**4**	**5**	**6**	**7**	**8**
Erectile function (IIEF-5)	−0.580^**^									
1: Age	−0.076^**^	0.056^*^								
2: Education	−0.097^**^	0.046^*^	−0.006							
3: Masturbate without porn	0.236^**^	−0.226^**^	−0.057^*^	−0.025						
4: Masturbate with porn	0.346^**^	−0.273^**^	−0.193^**^	0.029	0.355^**^					
5: Masturbate with partner present	0.123^**^	−0.167^**^	−0.091^**^	0.023	0.305^**^	0.293^**^				
6: Masturbate by partner manually stimulate	0.090^**^	−0.007	−0.120^**^	0.149^**^	0.099^**^	0.207^**^	0.140^**^			
7: Oral sex	−0.133^**^	0.104^**^	−0.034	0.208^**^	0.021	0.015	0.061^**^	0.608^**^		
8: Penile-vaginal sex	−0.325^**^	0.334^**^	0.003	0.077^**^	−0.143^**^	−0.199^**^	−0.080^**^	0.278^**^	0.403^**^	
9: Penile-anal sex	−0.129^**^	0.002	0.038	0.148^**^	0.044^*^	−0.041	0.094^**^	0.025	0.282^**^	0.081^**^

Abbreviations: CHEES, Checklist for Early Ejaculation Symptoms; IIEF-5, International Index of Erectile Function–5.

^a^Higher CHEES values suggest more ejaculatory problems. Higher IIEF-5 values suggest fewer erectile problems.

^^*^^ *P* < .05.

^^**^^ *P* < .01.

Table 8 shows the results of linear regression analyses between the frequency of different types of sex and ejaculatory function before controlling for age and education (*R*^2^ = 0.226, *F*_7,1968_ = 83.32, *P* < .001) and after (*R*^2^ = 0.231, *F*_7,1968_ = 66.744, *P* < .001). Table 9 shows the results of linear regression analyses between the frequency of different types of sex and erectile function before controlling for age and education (*R*^2^ = 0.173, *F*_7,1968_ = 59.899, *P* < .001) and after (*R*^2^ = 0.173, *F*_9,1966_ = 46.896, *P* < .001). The results indicated that a higher frequency of masturbation with or without porn and masturbation by a partner had an association with more ejaculatory problems. A higher frequency of oral sex, penile-vaginal sex, and penile-anal sex had an association with fewer ejaculatory problems. The frequency of all types of masturbation had an association with more erectile problems. The frequency of penile-vaginal sex had an association with more erectile problems.

**Table 8:** Linear regression analyses between the frequency of different types of sex and ejaculatory problems, before and after controlling for age and education.^a^

**CHEES (DV): controlled for**	***B***	**β**	***t***	***P* value**
Masturbate without porn	0.325	0.110	5.059	<.001
Age, education	0.313	0.107	4.890	<.001
Masturbate with porn	0.465	0.218	9.639	<.001
Age, education	0.468	0.219	9.621	<.001
Masturbate with partner present	−0.029	−0.007	−0.338	.735
Age, education	−0.030	−0.007	−0.350	.726
Masturbate by partner manually stimulate	0.280	0.187	7.073	<.001
Age, education	0.286	0.191	7.212	<.001
Oral sex	−0.190	−0.127	−4.555	<.001
Age, education	−0.173	−0.116	−4.145	<.001
Penile-vaginal sex	−0.555	−0.262	−11.667	<.001
Age, education	−0.557	−0.263	−11.726	<.001
Penile-anal sex	−0.178	−0.072	−3.380	.001
Age, education	−0.157	−0.063	−2.968	.003

Abbreviations: CHEES, Checklist for Early Ejaculation Symptoms; DV, dependent variable.

^a^Upper rows present the results of regression analyses before controlling for age and education; lower rows, after controlling for age and education.

**Table 9:** Linear regression analyses between the frequency of different types of sex and erectile function, before and after controlling for age and education.^a^

**IIEF-5 (DV): controlled for**	***B***	**β**	***t***	***P* value**
Masturbate without porn	−0.293	−0.108	−4.762	<.001
Age, education	−0.288	−0.106	−4.688	<.001
Masturbate with porn	−0.295	−0.149	−6.397	<.001
Age, education	−0.294	−0.149	−6.297	<.001
Masturbate with partner present	−0.216	−0.059	−2.657	.008
Age, education	−0.215	−0.059	−2.639	.008
Masturbate by partner manually stimulating	−0.085	−0.062	−2.252	.024
Age, education	−0.087	−0.063	−2.280	.023
Oral sex	0.057	0.041	1.437	.151
Age, education	0.050	0.036	1.257	.209
Penile-vaginal sex	0.564	0.287	12.382	<.001
Age, education	0.565	0.288	12.399	<.001
Penile-anal sex	−0.062	−0.027	−1.230	.219
Age, education	−0.071	−0.031	−1.400	.162

Abbreviations: DV, dependent variable; IIEF-5, International Index of Erectile Function–5.

^a^Upper rows present the results of regression analyses before controlling for age and education; lower rows, after controlling for age and education.

Table 10 shows the results of correlation analyses among the ELTs of different types of sex. The result indicated that longer ELTs during masturbation had significant positive associations with longer ELTs for partnered sex.

**Table 10:** Correlations among the ejaculatory latency times of different types of sex.^a^

**Ejaculatory latency time**	**Masturbate without porn**	**1**	**2**	**3**	**4**	**5**
1: Masturbate with porn	0.678^**^ (277)					
2: Masturbate with partner present	0.342^**^ (109)	0.584^**^ (171)				
3: Masturbate by partner manually stimulating	0.412^**^ (292)	0.384^**^ (652)	0.506^**^ (206)			
4: Oral sex	0.327^**^ (230)	0.293^**^ (501)	0.424^**^ (171)	0.540^**^ (946)		
5: Penile-vaginal sex	0.381^**^ (333)	0.411^**^ (739)	0.258^**^ (216)	0.394^**^ (1254)	0.455^**^ (1133)	
6: Penile-anal sex	0.135 (84)	0.208^*^ (124)	0.262^*^ (69)	0.279^**^ (191)	0.452^**^ (246)	0.564^**^ (289)

^a^Values in parentheses indicate sample sizes, which varied for these analyses, as not all participants had engaged in all types of sexual activities during the period of interest and therefore were not able to report ejaculation latency times.

^*^ *P* < .05.

^**^ *P* < .01.

Table 11 shows the associations of cardiovascular disease (have vs not) and smoking status (smoke or not) with erectile and ejaculatory problems, with the results based on *t*-test and partial correlation analyses after controlling for cardiovascular disease and smoking status. There were 66 (3.3%) participants who had any history of cardiovascular disease and 1910 (96.7%) who had not. There were 718 (36.3%) participants who smoked and 1258 (63.7%) who did not. The *t*-tests indicated that participants with cardiovascular disease and those who smoked had more ejaculatory problems (*t*_1974_ = −6.684, *P* < .001; *t*_1974_ = −4.937, *P* < .001) and erectile problems (*t*_1974_ = 7.468, *P* < .001; *t*_1974_ = 2.881, *P* < .01). After controlling for cardiovascular disease and smoking status, there was still a positive association between PE and ED symptoms.

**Table 11:** Descriptive data of the cardiovascular disease and smoking status groups.^a^

			**CHEES**		**IIEF-5**		
**Group**	**No.**	**%**	**Mean**	**SD**	**Mean**	**SD**	**Controlling for confounding**
Partial correlation							−0.568^***^
No cardiovascular	1910	96.7	9.318	2.737	21.906	2.518	
Cardiovascular	66	3.3	11.626	3.615	19.515	3.509	
No smoking	1258	63.7	9.162	2.508	21.953	2.299	
Smoking	718	36.3	9.805	3.211	21.605	3.028	

Abbreviations: CHEES, Checklist for Early Ejaculation Symptoms; IIEF-5, International Index of Erectile Function–5.

^a^Higher CHEES values suggest more ejaculatory problems. Higher IIEF-5 values suggest fewer erectile problems.

^***^ *P* < .001.

## Discussion

The present study investigated the prevalence of PE and its association with ED, demographic variables, sexual experience, and the frequency of different types of sex among Chinese men in the urban setting of Shanghai.

This is the first study to investigate the prevalence of PE with the CHEES in China. The results indicated that the prevalence of PE was almost 3.0%, which is less than in previous Chinese studies.^28^^,^^42^ However, just 7 (0.4%) participants had scores indicating a strong likelihood of PE and reported that their ELT was <1 minute, which was also less than in previous studies.^1^^,^^28^ In terms of the IELT, the prevalence of participants who self-reported that their IELT was within 1 minute (2.9%) was consistent with previous studies conducted in different countries.^48^^,^^49^ Yet, only 16 (0.8%) reported an IELT <1 minute when answering item 5 of CHEES. We think that the reason may be that the time span of the 2 questions was different: the former was about the entire sexual life; the latter was about the past 6 months. If men have a different sexual experience in the past 6 months as compared with before, there would be a difference between the answers.

The prevalence of ED was almost 35%, which is similar to a study in Korea.^50^ However, the prevalence was less than in a meta-analytic study among Chinese men^21^ but higher than the prevalence in some Western samples,^51^ which is consistent with a recent review.^52^ A logical reason is that the average age of the men in this study was lower than in previous samples with a higher prevalence, as ED primarily affects men aged >40 years.^20^ Nevertheless, the prevalence is still quite high, suggesting that ED is a major sexual problem among relatively young Chinese urban men as well.

The association between PE and ED was positive and strong, which is consistent with previous research.^22–24^^,^^53^ Men with more erectile problems seem to have more ejaculatory problems. Even though the result may not seem surprising, it is not immediately clear why this connection exists. However, the autonomous nervous system control of erection and ejaculation responses may provide an answer. The balance and timing of the sympathetic and parasympathetic nervous systems are crucial for a well-functioning male sexual response.^54^ First, in the erection phase, the parasympathetic nervous system activates the relaxation of muscles, otherwise limiting the flow of blood into the penis. In the ejaculation phase, the sympathetic nervous system facilitates the emission of seminal fluid.^54^ In the case of autonomic imbalance, sympathetic overactivity might inhibit erection and result in fast ejaculation. This may result in men with ED having more ejaculatory dysfunction as well.

Men with ED report more symptoms of sexual distress, depression,^19^^,^^55^ and sexual performance anxiety.^30^ Sexual distress^56^ and sexual performance anxiety^57^ during partnered sex could interfere with the parasympathetic facilitation of erection and result in an overactive sympathetic nervous system,^58–60^ triggering a worse imbalance of autonomic nervous system activity, which may also be a cause of poorer ejaculatory function in men. In this way, a common pathway seems to be driving ED and PE symptoms, resulting in a positive correlation between them.

The regression analyses revealed that, as predicted, men with more previous sexual experience had fewer erectile and ejaculatory problems after controlling for age and education level effects. The explanation for this relationship may be that men with more previous sexual experience have less sexual performance anxiety, a risk factor for PE and ED^30^; therefore, they have fewer erectile and ejaculatory problems. However, the underlying mechanism of the association between sexual function and previous sexual experience could be that the men with better erectile and ejaculatory function have more self-confidence^7^^,^^18^ in sexual performance, resulting in approaching potential sexual partners more often. It is also possible that these 2 possible directions act in a loop where initially small effects are reinforced over time.

The regression analyses suggested that a lower frequency of masturbation and a higher frequency of penile-vaginal sex had an association with fewer symptoms of ED and PE after controlling for age and education level, which was consistent with a previous study.^61^ More erectile and ejaculatory problems may lead to sexual dissatisfaction^11^^,^^18^ when men have penile-vaginal sex with their female partners. In addition, their female partners may be dissatisfied with the sexual relationship.^13^ As a consequence, men with PE and ED would have more sexual distress related to penile-vaginal sex, resulting in decreased sexual pleasure during partnered sex. Therefore, they might engage in more frequent masturbation as compensation.^36–38^

The results showed that the frequency of masturbating while the partner is present and being manually stimulated by one’s partner was associated with worse erectile functioning. A possible explanation for this association is that men with erectile difficulties might need additional physical stimulation to achieve a sufficiently hard erection for penetrative sex.

There were positive associations among the ELTs of masturbation, penile-vaginal sex, and other types of partnered sex (ie, oral, anal). The robust positive correlations among the ELTs of the different types of partnered sex were consistent with some previous research.^34^ According to the definition of PE,^1^ the symptoms of PE occur only in the partner sex context. However, our results suggest that the symptoms of PE may also occur during masturbation, even though it was previously found that men experienced less psychological stress during masturbation.^32^ Our results therefore suggest that the ELTs during different types of sex result from common, perhaps physiologic, factors. These could, for instance, include genetic influences^62^^,^^63^ or be related to a chronic imbalance of autonomic nervous activity.

Surprisingly, older men had fewer ejaculatory and erectile problems in the current sample. With increased age, many men would have more sexual experiences, which may be associated with decreased sexual performance anxiety, a known risk factor for PE and ED.^30^ Therefore, they may have fewer ejaculatory problems. However, the association between higher age and fewer erectile problems was inconsistent with previous studies.^21^^,^^24^ We think that the reason for this somewhat surprising result may be that the age range of the men in the present study was younger than in many previous studies that have examined ED. Given that the symptoms of ED primarily occur in men aged >40 years,^20^ the results are not so surprising.

In the current sample, men with higher educational levels had fewer ejaculatory and erectile problems, which is consistent with the previous study.^18^^,^^64^ We assume that the underlying reason may be that men with higher educational levels would put more emphasis on their female partners’ sexual pleasure and have more sexual communication that discusses and addresses sexual issues, especially for sexual problems with their partners, leading to more sexual satisfaction in an intimate relationship.^65^^,^^66^ For example, the men would aim to delay ejaculation to facilitate the sexual pleasure of their partners. The result indicated that, as predicted, men with cardiovascular disease and smoking habits had more ejaculatory and erectile problems, consistent with previous studies.^24^^,^^28^

## Conclusion

Men’s sexual experiences have an effect on their sexual function, which in turn affects their sexual activity.

### Limitations and future direction

There are some limitations to the study. First, this is the first time that PE has been investigated with the CHEES that includes self-reported measurements of ELT in a large Chinese sample. There may be a limitation to the validity of the CHEES and the reliability of such self-reports. Stopwatch IELT measurement can be considered an objective measurement. Importantly, stopwatch ILET has a strong correlation (*r* = 0.512, *P* < .001) with self-reported ILET measurement,^67^ suggesting that using self-reports is an acceptable approach, considering that measuring IELT may affect partnered sex through distraction and stress. Future studies should nevertheless consider additional measurements. Next, this study did not consider all the risk factors of PE and ED—for example, hypertension and obesity.^24^ Future studies should include these other risk factors. Last but not least, the age of the sample was relatively young. Future studies should also include more older men.

## Funding

The original data collections were funded by an internal NYU Shanghai grant to the last author.

## Conflicts of interest

No conflicts of interest.
